# Patient-reported outcomes among patients using exenatide twice daily or insulin in clinical practice in six European countries: the CHOICE prospective observational study

**DOI:** 10.1186/1477-7525-11-217

**Published:** 2013-12-26

**Authors:** Matthew Reaney, Chantal Mathieu, Claes-Göran Östenson, Stephan Matthaei, Thure Krarup, Jacek Kiljański, Carole Salaun-Martin, Hélène Sapin, Michael Theodorakis, Bruno Guerci

**Affiliations:** 1 Eli Lilly, Windlesham, Surrey, UK; 2 Department of Endocrinology, UZ Gasthuisberg, Leuven, Belgium; 3 Department of Molecular Medicine and Surgery, Karolinska Institutet, Stockholm, Sweden; 4 Diabetes-Center Quakenbrück, Quakenbrück, Germany; 5 Department of Endocrinology I, Bispebjerg Hospital, Copenhagen, Denmark; 6 Eli Lilly, Warsaw, Poland; 7 Eli Lilly, Neuilly Cedex, France; 8 Department of Clinical Therapeutics, University of Athens School of Medicine, Athens, Greece; 9 Diabetologie, Maladies Metaboliques & Nutrition, Hôpital Brabois, CHU de Nancy, et CIC Inserm, ILCV, 54500 Vandoeuvre Lès Nancy, France

**Keywords:** Diabetes mellitus, Type 2, Exenatide, Insulin, Injectable therapy, Patient-reported outcomes

## Abstract

**Background:**

Improvements in the clinical condition of patients with type 2 diabetes are often accompanied by improvements in health-related quality of life and other patient-reported outcomes (PROs), but data assessing injectable treatment initiation from the patient’s perspective in routine clinical practice are lacking. We examined PROs in patients initiating injectable treatment in the CHOICE (CHanges to treatment and Outcomes in patients with type 2 diabetes initiating InjeCtablE therapy) study.

**Methods:**

CHOICE was a 24-month, prospective observational study conducted in six European countries. Patients initiated exenatide twice daily (BID) or insulin based on a physician’s clinical judgement. Clinical and PRO data were collected at baseline (injectable therapy initiation) and after approximately 3, 6, 12, 18 and 24 months. The two treatment cohorts had different baseline characteristics; therefore, no statistical comparisons of endpoints between main cohorts were conducted.

**Results:**

There were 2388 patients eligible for analysis (exenatide BID cohort, n = 1114; insulin cohort, n = 1274). Mean positive changes in Impact of Weight on Quality of Life-Lite (IWQOL-Lite) total score and EuroQoL5-Dimension (EQ-5D) index and visual analogue scale (VAS) scores were observed in both cohorts with most changes observed during the first 6 months after injectable therapy initiation. Patients who experienced weight loss (≥1 kg) at 24 months appeared to have higher mean improvements in IWQOL-Lite total score than did patients with weight gain or no weight change. Patients who met the composite clinical endpoint of glycated haemoglobin (HbA1c) <7.0%, no weight gain (≤1 kg) and no hypoglycaemia generally experienced higher mean improvements in EQ-5D index and VAS scores (compared with patients who did not meet this endpoint) and Diabetes Health Profile-18 scores (versus the main cohorts). High levels of missing data were observed for all PRO measures in both cohorts compared with those for clinical outcomes.

**Conclusions:**

These data from a clinical practice study support those from clinical trials, suggesting that PROs are not adversely affected, and may be improved, by injectable therapy initiation. PRO data may aid appropriate treatment selection for individual patients.

**Trial registration:**

ClinicalTrials.gov, NCT00635492

## Background

The increasing global prevalence of type 2 diabetes (T2DM) is accompanied by increased clinical and economic burden [1]. Achieving good metabolic control, including tight control of blood glucose, contributes to reducing the clinical, psychological, and economic burden of T2DM, and this requires that healthcare professionals and patients work together to achieve optimal treatment of this chronic disease [2-5]. The influence of physical and social factors on T2DM incidence and health outcomes is also receiving attention [6]. Patients with T2DM require systematic, individualised and progressive interventions involving different therapies that address the clinical and psychosocial aspects of their illness [2,3,6,7]. A comprehensive evaluation of healthcare should ascertain a patient’s expressed health needs [7], as patient understanding, engagement, and commitment to the prescribed treatment strategy is key to meeting treatment goals and reducing morbidity and mortality associated with T2DM [3]. A patient’s perception of how his or her condition or treatment affects his or her quality of life is an important consideration when making treatment decisions, and physicians should consider this information as well as clinical data when discussing the available options with their patients [3,8].

The health-related quality of life (HRQoL) of patients with diabetes is often impaired, compared with a population without diabetes, and can be affected by both clinical and psychosocial factors [8,9]. HRQoL is inversely correlated with diabetes severity [5,9,10], and improvements in the clinical condition of patients with diabetes, particularly T2DM, are often accompanied by improvements in HRQoL and some other related patient-reported outcomes (PROs), such as health status and psychological well-being [8,11-14]. Newer T2DM therapies such as glucagon-like peptide-1 (GLP-1) receptor agonists are typically associated with weight loss; these therapies have been associated with improvements in weight-related quality of life (i.e. quality of life pertaining to weight) [11] and may also demonstrate other benefits on relevant PROs. Improvements in PROs have been reported from several randomised clinical trials (RCTs) of exenatide twice daily (BID) [15-17]. Although RCTs provide valuable data regarding the efficacy of a drug in an ‘ideal’ setting, the populations and management approach used in RCTs may not reflect actual clinical practice, where treatment is more complex and diverse [18]. Prospective observational studies are therefore used to investigate effectiveness, i.e., how well drugs work under real world conditions subject to several sources of variation, including patient characteristics, comorbidities and concomitant medications. Naturalistic studies such as these, conducted with less structure than RCTs, for a longer duration of time, and with a larger sample size, may yield different findings [17] and enhance the evidence upon which the management of T2DM is based [3,19,20].

The aim of the analyses reported in this manuscript was to understand the patient’s perspective following initiation of injectable antidiabetes medication in routine clinical practice. PROs were examined using data from the CHOICE (CHanges to treatment and Outcomes in patients with type 2 diabetes initiating InjeCtablE therapy) study [21-24]. Exenatide BID and insulins were the only injectable treatments available for T2DM when this study commenced. Therefore, the study recruited patients initiating either exenatide BID or their first insulin regimen in routine clinical practice. Baseline patient characteristics [21] and clinical outcomes, healthcare resource use, and costs during the 24 months after initiation of injectable therapy in CHOICE have been reported elsewhere [22-24]. Understanding PROs following injectable therapy initiation will provide additional insight from the patient’s perspective that, together with clinical data, will help patients and clinicians to make better informed treatment decisions.

### Patients and methods

#### ***Study design and patients***

CHOICE (http://www.clinicaltrials.gov identifier NCT00635492) was a prospective, noninterventional observational study that recruited patients from six European countries (Belgium, Denmark, France, Germany, Greece, and Sweden) between January 2008 and October 2009. Patients aged ≥18 years and initiating their first injectable antidiabetes therapy with exenatide BID or insulin for T2DM in routine clinical practice were included in the study. Patients were invited to participate in CHOICE only after the clinical decision had been made to initiate exenatide BID or insulin for the treatment of T2DM (in addition to any oral antidiabetes drugs required). Treatment choice (exenatide BID or insulin) was based on the clinical judgement of the patient’s physician. Patients gave written informed consent for the use of their data and appropriate ethical review board approval was obtained from the Ethics Committee of the State Medical Association (Frankfurt, Germany), the Regional Ethical Review Board (Stockholm, Sweden), and the Medical Ethics Committee of University Hospitals Leuven (Leuven, Belgium). Further details on the design of the CHOICE study have been reported previously [21].

The primary endpoint of CHOICE was the time from the initiation of initial injectable regimen (exenatide BID or insulin) to significant treatment change (see Mathieu et al. [22] for definitions). The study also aimed to describe the characteristics of patients with T2DM initiated on injectable therapy [21], the factors associated with changes to treatment, clinical outcomes, PROs, and the healthcare resource use observed over 24 months.

Data were collected from each patient at baseline (initiation of injectable therapy) and at follow-up visits when they occurred as part of clinical practice, approximately 3, 6, 12, 18, and 24 months (all ± 6 weeks) after baseline.

#### ***Measures***

PRO endpoints were measured using standardised and validated questionnaires. Weight-related quality of life was assessed using the Impact of Weight on Quality of Life-Lite questionnaire (IWQOL-Lite), a 31-item scale that assesses the domains of physical function, self-esteem, sexual life, public distress, and work [25]. Response categories range from 1 = “never true” to 5 = “always true”. Total scores are transformed in a linear manner to IWQOL-Lite “standardised scores”, ranging from 0 to 100, with higher standardised scores indicating better quality of life [26].

Health status was measured using the generic EuroQol-5-Dimension (EQ-5D) instrument [27] [three-level (3L) version]. In the EQ-5D, patients are asked to report their level of functioning in five dimensions (mobility, self-care, usual activities, pain/discomfort, anxiety/depression), with each dimension assessed by one item with three response choices (no problems, some problems, severe problems). Responses to the five items are used to derive an overall health index score (using the UK weighting) with a possible range from -0.594 to 1.0, where 0 represents death and 1.0 represents a perfect health state (values below zero represent a state considered to be “worse than death”) [28]. In addition, the EQ-5D contains a single item visual analogue scale (VAS) on which patients rate their current health state on a scale ranging from 0 (worst imaginable health state) to 100 (best imaginable health state).

HRQoL was measured using the Diabetes Health Profile-18 instrument (DHP-18), an 18-item diabetes-specific questionnaire with three domains: barriers to activity, disinhibited eating, and psychological distress [29]. Each question is scored using a 4-point Likert-type scale ranging from 0 to 3, and subscale raw scores can be transformed to a common score range of 0–100 with 0 representing no dysfunction.

Emotional distress was measured using the Hospital Anxiety and Depression Scale (HADS), a 14-item questionnaire (seven items each for anxiety or depression) for which each item is answered on a four-point scale (0–3) [30]. Raw subscale scores for anxiety and depression are calculated by adding all item scores together for a maximum possible score of 21. The developers provide clinically defined cut-off points to indicate whether a patient is “within the normal range” (score of 0–7), or in a “mildly” (8–10), “moderately” (11–14), or “severely” (15–21) disordered state.

### Analysis

#### ***Sample size justification***

The sample size for CHOICE was based on the primary endpoint of time to first significant treatment change [21,22]; as such the study was not powered to assess changes in PROs. Sample size was calculated using a Monte-Carlo simulation, assuming annual patient dropout rates of 10% to 15% and a median time to significant treatment change of 9.0 months for the exenatide BID cohort and 8.6 months for the insulin cohort [31,32]. Based on this, the study aimed to recruit a maximum of 800 patients per country/country group. The insulin cohort was to be larger than the exenatide BID cohort (60% vs. 40% of patients) because of the greater variability in the insulin cohort (linked to use of different insulin regimens).

### Statistical analysis

All patients who provided consent to release information, fulfilled study entry criteria, had a case report form summary page signed by an investigator and had at least one post-baseline assessment were included in the analyses (“eligible patients”). Due to the observational nature of this study, patients who violated the study description or who discontinued early from the study were included in the analyses.

As anticipated, analysis of the baseline data indicated that the two treatment cohorts comprised substantially different patient populations [21]. As a consequence, statistical comparisons of endpoints between the two main cohorts were not conducted and analyses of PRO endpoints are descriptive only.

Analyses of the PRO endpoints were conducted using available data from all eligible patients; data collected until study discontinuation were analysed according to the cohort (exenatide BID or insulin) that patients were placed in at baseline (“initiators” analysis). Item, domain, and total scores were summarised, as relevant, using frequency distribution and descriptive statistics. Absolute numbers and percentages (based on the number of patients with visits at the respective time point, as these patients had the opportunity to provide data) were given for categorical variables. Patients in Germany were not asked to complete the HADS or IWQOL-Lite questionnaires (due to general ethical concerns in Germany that patients may potentially feel overburdened when asked to complete several questionnaires), so percentage data for these measures are based on the number of patients with visits, excluding those patients in Germany. Item-level missing data were dealt with according to the instructions from the PRO instrument developers. The potential relationship between various clinical parameters and relevant PROs was also examined, for example, whether IWQOL-Lite scores were associated with weight loss or gain.

Cox regression models were performed post hoc to investigate the association of baseline characteristics with time to achieving the clinically relevant composite endpoint of HbA1c <7.0%, no weight gain (≤1 kg), and no hypoglycaemia [33].

### Interpretation of PRO data

To assist in interpreting PRO scores, a meaningful change in individual patient scores needs to be identified [34,35]. The proportion of patients meeting minimally important changes (MICs) in individual PRO scores was determined using published recommendations where available. Therefore, a change from baseline of >0.03 on the EQ-5D index [36], a change of >3.0 on the EQ VAS [36], and change in DHP-18 scores for barriers to activities of >5.29, disinhibited eating of >2.80 and psychological distress of >4.87 [37] constituted a MIC.

As there are no published MICs for the HADS (except in patients with chronic obstructive pulmonary disease [38]) and those for the IWQOL-Lite [39] considered participants enrolled in weight loss studies/programmes only (it was not reported whether any participants had diabetes), the distributions of responses to these questionnaires were calculated using a cumulative distribution function (CDF). This shows all magnitudes of change across the entire study population and the proportion of patients at each point along the scale score continuum who experience change at that level or lower [40]. This allows the reader to calculate the percentage of responders at each value of the change score and evaluate the consistency of changes across different response thresholds.

## Results

### Clinical endpoints in CHOICE

A total of 2515 patients were recruited from 322 investigator sites, mainly secondary care sites. Overall, there were 2388 eligible patients in this analysis; 1114 in the exenatide BID cohort and 1274 in the insulin cohort. Visit attendance decreased over time to 873 exenatide BID (78.4%) and 1025 insulin (80.5%) patients at the 24-month visit. Over 24 months, 23.5% of patients discontinued the study: lost to follow-up was the primary reason in both cohorts (about 13.5% of each cohort), although 7.4% of exenatide BID and 3.5% of insulin patients discontinued due to subject decision [22].

Significant differences were observed in the baseline patient characteristics of the exenatide BID and insulin treatment cohorts (see Matthaei et al. [21]). Statistical comparisons of clinical and PRO endpoints between the two main cohorts were therefore not conducted.

A total of 470 patients from the exenatide BID cohort (42.2%) and 459 patients from the insulin cohort (36.0%) had a significant treatment change [22] during the study. In the exenatide BID cohort, 74.3% of the first significant treatment changes were discontinuations of initial injectable therapy, with the rest comprising the addition of oral or injectable antidiabetes medication to their exenatide BID regimen. Overall, the most common first significant treatment change for insulin patients was the addition of a new oral or injectable medication (58.2% of first significant treatment changes). Discontinuations of ≥1 insulin initiated at baseline accounted for 24.2% of the first significant treatment changes for patients in the insulin cohort.

During the study, 393 patients in the exenatide BID cohort (35.3%) and 155 patients in the insulin cohort (12.2%) discontinued their initial injectable therapy. The most common reason for such discontinuation in both cohorts was inadequate response [170 patients in the exenatide BID cohort (15.3%) and 87 patients in the insulin cohort (6.8%)]. Adverse events were cited as the reason for treatment discontinuation for 91 patients in the exenatide BID cohort (8.2%) and 11 patients in the insulin cohort (0.9%) [22].

Table 1 presents the main clinical data at baseline and 24 months and a full description of the clinical data has been reported previously [22]. Glycaemic control improved in both the exenatide BID and insulin cohorts. A mean weight loss was seen in the exenatide BID cohort, whereas a mean weight gain was seen in the insulin cohort. Gastrointestinal (GI) events were experienced by 30.8% of the exenatide BID cohort and 5.3% of the insulin cohort. The proportion of exenatide BID patients with GI events was higher in the first 6 months of the study (26.2% of patients with data) than in subsequent 6-month periods (<8% of patients with data).

**Table 1:** Baseline characteristics and 24-month clinical outcomes: patients initiated on exenatide twice daily (BID) or insulin

**Variable**	**Exenatide BID**		**Insulin**	
	**Baseline**		**Baseline**	
	**(N = 1114)**^**a**^		**(N = 1274)**^**a**^	
Male, *n* (%)	n = 1114		n = 1274	
	598 (53.7)		733 (57.5)	
Age, years	n = 1114		n = 1274	
	58.1 (10.1)		63.7 (10.9)	
Time since diabetes diagnosis, years	n = 1105		n = 1263	
	8.2 (5.7)		9.8 (7.3)	
Diabetes complications, *n* (%)	n = 1114		n = 1274	
≥1 macrovascular complication	200 (18.0)		320 (25.1)	
≥1 microvascular complication	164 (14.7)		263 (20.6)	
	**Baseline**	**24 months**	**Baseline**	**24 months**
	**(N = 1114)**^**a**^	**(N = 873)**^**a**^	**(N = 1274)**^**a**^	**(N = 1025)**^**a**^
Weight, kg	n = 1112	n = 810	n = 1270	n = 947
	101.2 (21.7)	98.3 (21.3)	84.2 (17.6)	86.7 (17.8)
BMI, kg/m^2^	n = 1100	n = 805	n = 1265	n = 942
	35.3 (6.6)	34.2 (6.4)	29.7 (5.4)	30.6 (5.5)
Blood pressure, mmHg	n = 1103	n = 769	n = 1259	n = 895
Systolic	137.7 (16.5)	134.8 (15.2)	137.4 (17.4)	133.9 (15.3)
Diastolic	81.7 (9.6)	78.7 (9.7)	80.2 (9.9)	78.0 (8.8)
HbA1c,%	n = 1087	n = 812	n = 1245	n = 944
	8.4 (1.4)^b^	7.3 (1.2)	9.2 (1.9)^b^	7.3 (1.1)
Patients with HbA1c <7%, *n* (%)	n = 1087	n = 705	n = 1245	n = 871
	128 (11.8)	258 (36.6)^c^	75 (6.0)	333 (38.2)^c^
No. of OADs used, *n* (%)	n = 1114	n = 873	n = 1274	n = 1025
0	76 (6.8)	94 (10.8)	333 (26.1)	304 (29.7)
1	499 (44.8)	375 (43.0)	574 (45.1)	473 (46.1)
2	491 (44.1)	341 (39.1)	341 (26.8)	220 (21.5)
≥3	48 (4.3)	63 (7.2)	26 (2.0)	28 (2.7)
Patients with ≥1 hypoglycaemic event, *n* (%)^d^	n = 1112	n = 1061	n = 1274	n = 1221
	59 (5.3)	195 (18.4)	56 (4.4)	449 (36.8)
Patients with ≥1 GI symptom, *n* (%)	n = 1113	n = 1060	n = 1273	n = 1219
	72 (6.5)	327 (30.8)^e^	47 (3.7)	64 (5.3)^e^
Patients achieving lipid targets, *n* (%)				
HDL-C >50 mg/dl	n = 989	n = 651	n = 1083	n = 737
	287 (29.0)	234 (35.9)	336 (31.0)	278 (37.7)
LDL-C <100 mg/dl	n = 967	n = 635	n = 1055	n = 729
	420 (43.4)	292 (46.0)	384 (36.4)	307 (42.1)
Triglycerides <150 mg/dl	n = 1005	n = 659	n = 1118	n = 753
	362 (36.0)	326 (49.5)	450 (40.3)	417 (55.4)
HDL-C >50, LDL-C <100 & Triglycerides <150 mg/dl	n = 1012	n = 659	n = 1113	n = 748
	73 (7.2)	81 (12.3)	75 (6.7)	82 (11.0)

Continuous data are means (SD).

BID = twice daily; BMI = body mass index; HbA1c = glycated haemoglobin; OAD = oral antidiabetes drug; GI = gastrointestinal; HDL-C = high-density lipoprotein cholesterol; LDL-C = low-density lipoprotein cholesterol; SD = standard deviation.

^a^N numbers represent the number of patients who attended a baseline or 24-month visit. Percentages are based on the number of patients with data.

^b^Most recent in previous 3 months.

^c^Data from the subgroup of 959 patients in the exenatide BID initiators group and 1170 patients in the insulin initiators group with baseline HbA1c ≥7.0% at baseline (254 and 299 patients, respectively, had missing data).

^d^Patient recall: baseline = past 3 months; 24 months = patients recalled events at each visit (between baseline and 24 months) that they had experienced since their previous visit.

^e^Patient recall: Baseline = past 4 weeks; 24 months = patients recalled symptoms at each visit (between baseline and 24 months) that they had experienced since their previous visit.

### Patient-reported outcomes

Table 2 summarises the baseline PRO scores for patients in the exenatide BID and insulin cohorts. High levels of missing data and large standard deviations (SDs) were noted for all PRO measures in both cohorts.

**Table 2:** Patient-reported outcome (PRO) scores at baseline

**PRO**	**Exenatide BID**	**Insulin**
	**N = 1114**^**a**^	**N = 1274**^**a**^
IWQOL-Lite*		
Total score	n = 715	n = 817
	77.25 (19.41)	84.49 (17.37)
Subscales	n = 648–707^b^	n = 725–812^b^
Physical function	69.35 (23.68)	77.84 (21.03)
Self-esteem	76.31 (26.68)	86.47 (21.41)
Sexual life	76.25 (28.50)	82.54 (26.81)
Public distress	89.25 (18.77)	93.60 (15.51)
Work	85.50 (20.15)	90.25 (18.09)
**EQ-5D**		
Index score**	n = 1079	n = 1242
	0.73 (0.27)	0.71 (0.27)
Subscales	n = 1086–1090	n = 1249–1254
Number (%)^c^ of patients who reported at least some problems with:		
Mobility	322 (28.9)	442 (34.7)
Self-care	76 (6.8)	125 (9.8)
Usual activities	232 (20.8)	301 (23.6)
Pain/discomfort	588 (52.8)	679 (53.3)
Anxiety/depression	523 (46.9)	607 (47.6)
VAS score++	n = 1063	n = 1225
	64.63 (17.94)	63.77 (19.13)
DHP-18***		
Barriers to activity	n = 1090	n = 1251
	30.95 (21.41)	29.45 (19.41)
Disinhibited eating	n = 1089	n = 1253
	45.88 (21.75)	38.35 (21.65)
Psychological distress	n = 1086	n = 1245
	29.18 (21.34)	26.53 (21.07)
**HADS**^**+ **^**anxiety**	n = 692	n = 794
	6.38 (4.40)	6.96 (4.59)
**HADS**^**+ **^**depression**	n = 695	n = 787
	5.44 (4.09)	6.04 (4.35)

Data are presented as mean (SD), unless otherwise stated.

PRO = patient-reported outcome; BID = twice daily, IWQOL-Lite = Impact of Weight on Quality of Life-Lite, EQ-5D = EuroQoL-5D, VAS = visual analogue scale, DHP-18 = Diabetes Health Profile-18, HADS = Hospital Anxiety and Depression Scale.

^a^N represents the number of patients who attended a baseline visit. Percentages are based on N. The number of patients with data for each endpoint or group of endpoints (n) is presented above that endpoint or group of endpoints.

^b^For both cohorts, the lowest n number for the IWQOL-Lite subscales was for data for the sexual life subscale, the highest n number was for the physical function subscale.

^c^Percentages are based on the number of patients who attended the baseline visit.

*IWQOL-Lite standardised scores, range 0–100. Higher scores indicate higher quality of life. As IWQOL-Lite was not applied in Germany, the numbers of patients who attended a baseline visit and had the opportunity to provide IWQOL-Lite data were 730 (exenatide BID cohort) and 836 (insulin cohort).

**U.K.-specific coefficients and country-specific coefficients were used where available. Higher scores indicate better health status.

++Range 0–100. Higher scores indicate better health status.

***Standardised scores for subscales, range 0–100. Lower scores indicate better health-related quality of life.

+Raw scores for subscales, range 0–21. Lower scores indicate lower levels of emotional distress. As HADS Anxiety and Depression were not applied in Germany, the number of patients who attended a baseline visit and had the opportunity to provide HADS data were 730 (exenatide BID cohort) and 836 (insulin cohort).

#### ***IWQOL-Lite***

**Figure 1:**
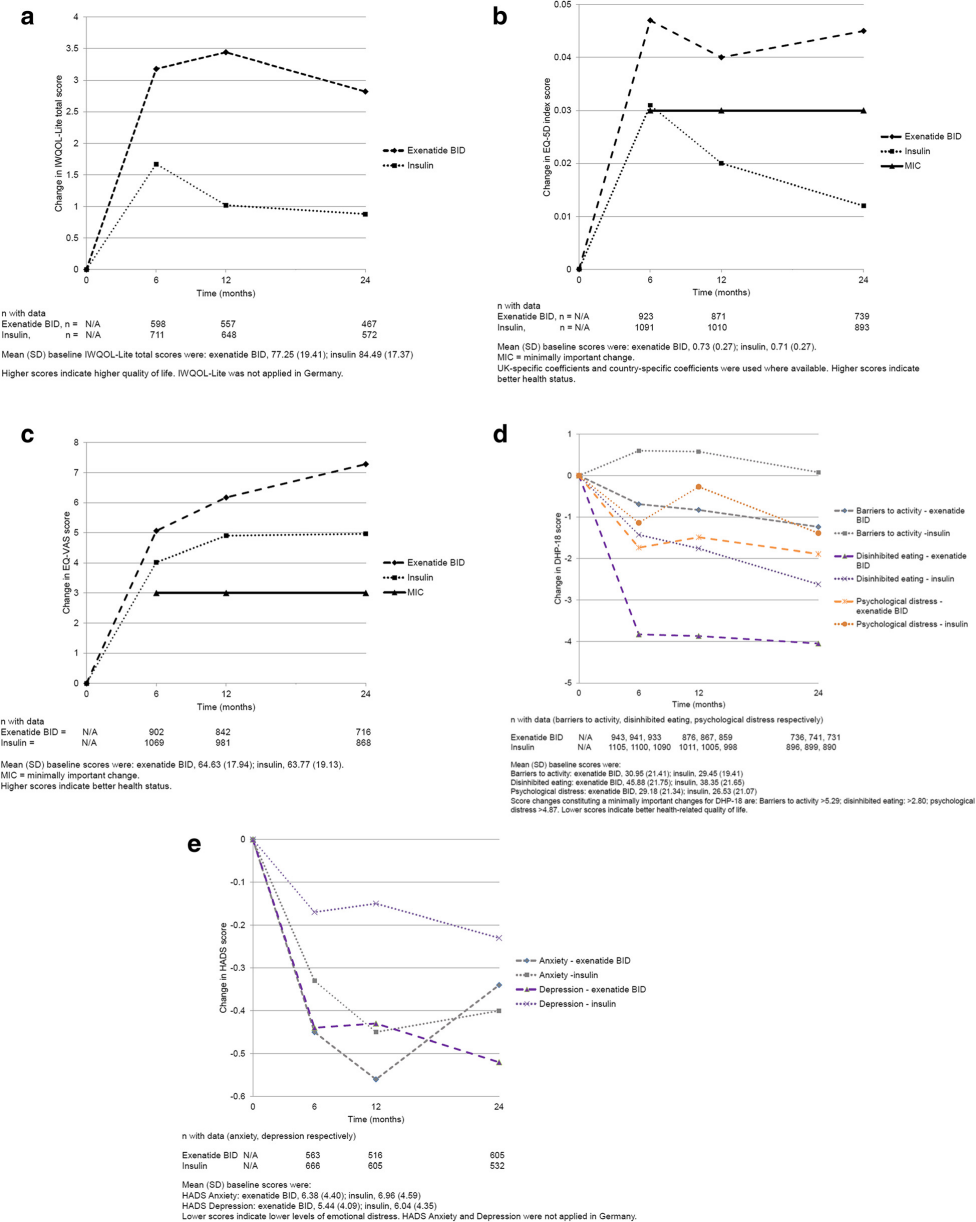
**Changes in patient-reported outcomes (PROs) over 24 months in the CHOICE study.** Changes from baseline in PRO measures over 24 months after initiation of exenatide twice daily (BID) or insulin are presented. The number of patients with change data at each time point is presented below the time point. **a)** Mean change in standardised Impact of Weight on Quality of Life-Lite (IWQOL-Lite) total score. **b)** Mean change in EuroQoL-5-Dimension (EQ-5D) index score. **c)** Mean change in EuroQoL-visual analogue scale (EQ-VAS) score. **d)** Mean changes in standardised Diabetes Health Profile-18 (DHP-18) scores. **e)** Mean change in Hospital Anxiety and Depression Scale (HADS) scores.

There was a mean positive change in overall IWQOL-Lite total score from baseline in both treatment cohorts during the first 6 months after initiation of injectable therapy (Figure 1a). Thereafter scores tended to plateau in the exenatide BID cohort and decrease in the insulin cohort, remaining above baseline levels throughout the 24-month study. When weight change (≥1 kg in either direction) at 24 months was considered, mean (SD) baseline IWQOL-Lite scores for patients who later had weight change (compared with baseline) at 24 months were: exenatide BID, weight loss 75.51 (19.64); weight gain 79.17 (18.63); no weight change 80.48 (18.00); insulin: weight loss 81.17 (19.41); weight gain 85.09 (16.57); no weight change 85.81 (17.42). Mean (SD) changes in IWQOL-Lite scores (according to weight change) from baseline to 24 months in the exenatide BID cohort were: weight loss +4.36 (13.90), weight gain -0.13 (12.34), no weight change +1.18 (13.32). Respective change values for the insulin cohort were: +2.98 (13.16), –0.04 (11.57), +1.61 (11.01).

**Figure 2:**
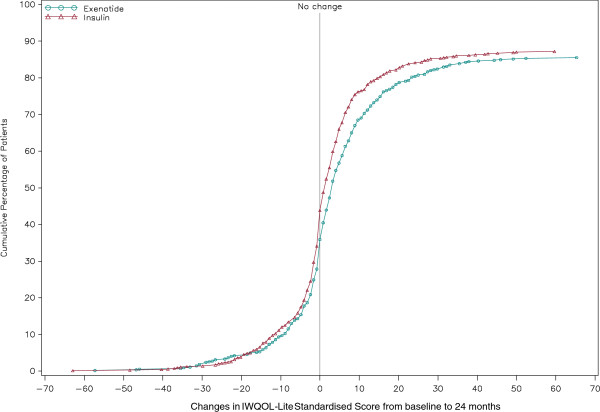
**CDF of IWQoL-Lite questionnaire total score change from baseline to 24 months.** CDF = cumulative distribution function; IWQOL-Lite = Impact of Weight on Quality of Life-Lite Higher scores indicate higher quality of life. IWQOL-Lite was not applied in Germany.

The CDF for the IWQOL-Lite total score (Figure 2) showed that 57.7% of exenatide BID and 53.0% of insulin patients reported no worsening in IWQOL-Lite scores at the 24-month visit (missing data overall: 13.6%).

#### ***EQ-5D***

The proportions of patients with visits (in both cohorts) who reported at least some problems with the single domains “mobility”, “self-care” and “usual activities” of the EQ-5D generally showed little change over the 24 months of the study (data not shown), although the percentage of patients overall with missing data for EQ-5D single domains increased from ~2% at baseline to ~12% at the 24-month visit. However, the proportions of patients reporting at least some problems with “pain/discomfort” were 52.8% at baseline and 43.4% at 24 months in the exenatide BID cohort and 53.3% at baseline and 47.4% at 24 months in the insulin cohort. The proportions of patients who reported at least some problems with “anxiety/depression” were 46.9% at baseline and 35.4% at 24 months in the exenatide BID cohort; respective values were 47.6% and 35.9% in the insulin cohort.

The greatest mean improvements in the EQ-5D index score were observed between baseline and 6 months for both cohorts (Figure 1b). Mean changes were above MIC for the exenatide BID cohort at all time points, and were above MIC only at 6 months in the insulin cohort; between baseline and 24 months, 32.0% of patients in the exenatide BID cohort and 27.3% of patients in the insulin cohort had improved EQ-5D index scores by more than the MIC (Table 3). Similarly, both cohorts experienced a mean increase in the EQ-VAS score over 24 months, with most change occurring between baseline and 6 months (Figure 1c). Mean changes in EQ-VAS scores were above the MIC at all time points for both cohorts; EQ-VAS scores had improved by more than the MIC for 47.4% of patients in the exenatide BID cohort and 44.7% of patients in the insulin cohort at 24 months (Table 3). Post hoc multivariate Cox regression models showed that baseline EQ-5D index values (VAS scores were not evaluated) were not significantly associated with time to achieving the composite endpoint proposed by Zinman et al. [33]. However, patients in both the exenatide BID and insulin cohorts who met the composite endpoint (n = 271 in the exenatide BID cohort, n = 144 in the insulin cohort) experienced numerically greater changes in mean (SD) EQ-5D index and VAS score after 24 months [exenatide BID: index: +0.08 (0.23), VAS: +9.01 (18.24); insulin: index: +0.02 (0.32), VAS: +7.57 (21.78)] than the respective group of patients who did not meet the composite endpoint [exenatide BID: index: +0.04 (0.24), VAS: +6.69 (16.69); insulin: index: +0.01 (0.26), VAS: +4.63 (16.95)].

**Table 3:** Patients with a change in PROs greater than the MIC from baseline to 24 months

**PRO**	**Exenatide BID**	**Insulin**
	(N = 873)*	(N = 1025)*
EQ-5D index score	n = 739	n = 893
n (%) improving by > MIC (0.03)	279 (32.0)	280 (27.3)
n (%) worsening by > MIC (0.03)	194 (22.2)	288 (28.1)
EQ-5D VAS score	n = 716	n = 868
n (%) improving by > MIC (3.0)	414 (47.4)	458 (44.7)
n (%) worsening by > MIC (3.0)	150 (17.2)	224 (21.9)
DHP-18 (standardised scores)		
Barriers to activity	n = 736	n = 896
n (%) improving by > MIC (5.29)	231 (26.5)	251 (24.5)
n (%) worsening by > MIC (5.29)	174 (19.9)	250 (24.4)
Disinhibited eating	n = 741	n = 899
n (%) improving by > MIC (2.80)	364 (41.7)	415 (40.5)
n (%) worsening by > MIC (2.80)	247 (28.3)	308 (30.0)
Psychological distress	n = 731	n = 890
n (%) improving by > MIC (4.87)	306 (35.1)	365 (35.6)
n (%) worsening by > MIC (4.87)	254 (29.1)	311 (30.3)

PRO = patient-reported outcome; MIC, minimally important change; BID = twice daily, EQ-5D = EuroQoL-5D, VAS = visual analogue scale, DHP-18 = Diabetes Health Profile-18.

*N numbers represent the number of patients who attended a 24-month visit. Percentages are based on N. The number of patients with data for each endpoint (n) is presented above each endpoint.

#### ***DHP-18***

Changes in DHP-18 scores over the 24-month study are presented in Figure 1d. Most changes were small and below their respective MIC, except for change in disinhibited eating at all time points for the exenatide BID cohort. The number of patients whose DHP-18 scores improved or worsened by more than the MIC at the 24-month visit is shown in Table 3.

In both cohorts, patients who met the composite endpoint had numerically lower (better) mean baseline scores compared with their respective total cohort for all DHP-18 parameters (data not shown; baseline scores for the total cohort are presented in Table 2). Patients who met the composite endpoint also generally experienced numerically greater DHP-18 score improvements over 24 months than those in the main cohort. The differences in DHP-18 score changes between patients who met the composite endpoint and the main cohorts were generally lower than the MIC, with the exception of disinhibited eating at 24 months in the exenatide BID cohort (data not shown).

#### ***HADS anxiety and depression***

Changes in HADS anxiety and depression scores are presented in Figure 1e.

At baseline, 59.2% of exenatide BID and 53.8% of insulin patients had responses “within the normal range” for anxiety. Responses in the “mildly”, “moderately” or “severely” disordered states were given by 18.2%, 13.2% and 4.2% of exenatide BID patients and 20.8%, 14.6%, and 5.7% of insulin patients, respectively. For depression, 66.2% of exenatide BID and 60.6% insulin patients had responses “within the normal range”. Responses in the “mildly”, “moderately” or “severely” disordered states were given by 16.3%, 10.0% and 2.7% of exenatide BID patients and 17.9%, 11.8% and 3.7% of insulin patients. The proportion of patients with responses within the normal range and in each disordered state generally decreased over the course of the study (data not shown), but changes were small and the amount of missing data increased (overall: anxiety 5.1% at baseline, 16.0% at 24 months; depression 5.4% at baseline, 17.4% at 24 months).

**Figure 3:**
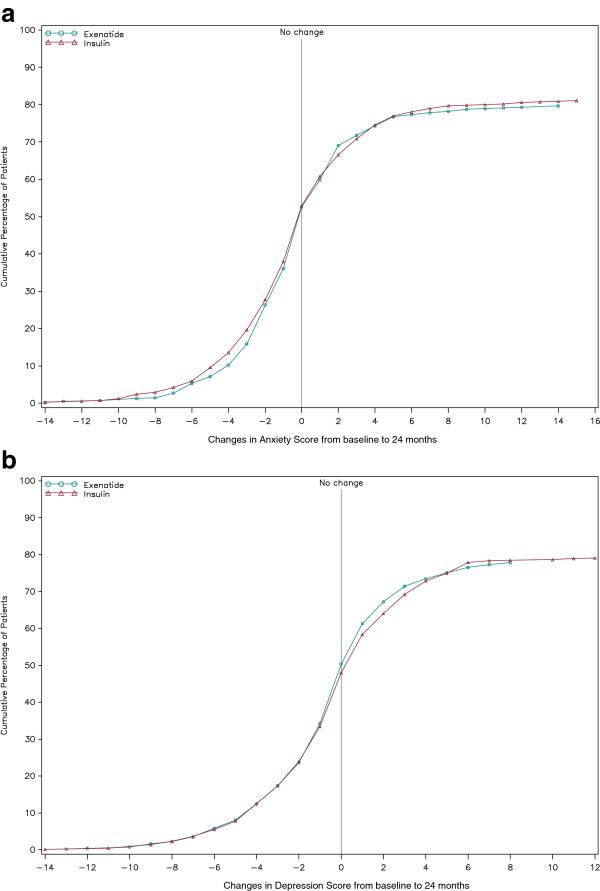
**CDF of HADS score change from baseline to 24 months. a)** CDF for HADS anxiety score change from baseline. **b)** CDF for HADS depression score change from baseline to 24 months. CDF = cumulative distribution function; HADS = Hospital Anxiety and Depression Scale Lower scores indicate lower levels of emotional distress. HADS Anxiety and Depression were not applied in Germany.

The CDFs for the HADS anxiety and depression scores (Figures 3a and b) indicate that 52.6% and 50.3% of exenatide BID patients and 52.9% and 48.0% of insulin patients reported no worsening in HADS anxiety and depression scores, respectively, at the 24-month visit (missing data overall: anxiety 19.6%; depression 21.5%).

## Discussion

The initiation of injectable therapy may represent an important milestone for the patient from both a clinical and personal point of view: in addition to the knowledge that their condition has progressed to requiring injectable therapy, the patient may be fearful of injections and side effects. CHOICE measured PROs at, and during the 24 months following, initiation of injectable therapy with exenatide BID or insulin. The data suggest that initiation of injectable therapy with either exenatide BID or insulin does not adversely affect weight-related quality of life, health status, HRQoL, or emotional distress, and may also be associated with improvements in some PRO measures. These PRO data support those from previous clinical trials [15-17] and help address an identified need for long-term prospective data to understand whether PRO benefits observed in trials of incretin therapy are realised in clinical practice [11]. Analysis of PRO data from the CHOICE study supports injectable therapy initiation, suggesting that, from the patients’ perspective, the disutility associated with daily injections [41,42] is offset by the clinical improvements observed. Consistent with clinical variables [22], most change in PRO measures was seen in the first 6 months.

Heavier body weight has been associated with disutility in patients with T2DM, and lower body weight with added utility [41]. In CHOICE, patients who later achieved weight loss (≥1 kg) appeared to have poorer weight-related quality of life at baseline (i.e. before injectable therapy initiation), compared with those who experienced either no weight change or weight gain, suggesting that poor weight-related quality of life could improve motivation to lose weight. As expected, weight-related quality of life then appeared to be affected by whether or not patients gained or lost weight (≥1 kg): those who experienced weight loss appeared to have higher mean IWQOL-Lite score changes than those with no weight change or weight gain, indicating an improvement in weight-related quality of life as well as the clinical benefits of weight loss for these patients. However, although patients in the exenatide BID cohort experienced a mean weight loss and those in the insulin cohort experienced a mean weight gain, an overall mean increase in IWQOL-Lite score was observed for both cohorts during the study. The improvement in IWQOL-Lite score following the initiation of exenatide BID in CHOICE is in agreement with 12-month results from the U.S. exenatide BID observational study (ExOS) [43].

EQ-5D index and VAS scores generally improved throughout the CHOICE study. These changes are encouraging, given that patients with T2DM in the longitudinal US Study to Help Improve Early evaluation and management of risk factors Leading to Diabetes (SHIELD) had a significantly greater decline in EQ-5D index scores over 5 years, compared with people without diabetes, and this decline was greater in patients with T2DM complications than in those without complications [44]. The proportion of patients in both cohorts of the CHOICE study reporting at least some problems with “anxiety/depression” numerically decreased over 24 months according to EQ-5D, and small mean improvements were observed in anxiety and depression according to the HADS. However, both these observations were confounded by increasing proportions of missing data from baseline to 24 months. At the 24-month visit, around half of patients in both cohorts reported no worsening in HADS anxiety and depression scores, although around 20% of patients had missing data for this analysis. Depression is a recognised problem in patients with T2DM [45], and both EQ-5D and HADS data may be important in monitoring this issue. EQ-5D data are required by reimbursement agencies to make decisions but HADS may be more relevant to clinical practice.

Although post hoc analyses revealed that baseline EQ-5D index score (VAS was not included in these analyses) was not significantly associated with time to achieving the composite clinical endpoint (HbA1c <7.0%, no weight gain (≤1 kg), and no hypoglycaemia [33]), greater mean changes in EQ-5D index and VAS scores were observed for patients in both cohorts who met this composite endpoint during the study than for those who did not meet this endpoint. This suggests that achievement of meaningful clinical improvement (following injectable treatment initiation) may result in improved health status and that better health status at baseline may not influence the likelihood of patients later achieving meaningful clinical improvement.

All of the previously discussed PROs are generic and are used across a range of clinical conditions. Generic instruments include items that may be irrelevant and/or do not specifically enhance our understanding of the impact of diabetes, and they exclude domains that are likely to be of great relevance [46]. Diabetes-specific instruments do not allow comparison with other conditions, but they are likely to be “more sensitive to change and responsive to subgroup differences than a generic instrument” [47]. The DHP-18 is a diabetes-specific PRO measure and was used to examine the potential impact of injectable therapy initiation on diabetes-related HRQoL. Most changes in DHP-18 scores were below the relevant MICs, with the exception of improved disinhibited eating at all time points for the exenatide BID cohort. This improvement was achieved after 6 months and maintained throughout the study. It is interesting that the main improvement in disinhibited eating in the exenatide BID cohort was observed during the first 6 months. Although the proportion of exenatide BID patients experiencing GI events decreased as the study progressed, these data suggest that the improvement in disinhibited eating was not completely offset by GI events. Patients who met the composite clinical endpoint appeared to have higher DHP-18 score changes over 24 months than was observed in the total cohort.

These data add to a growing body of clinical evidence regarding the initiation of injectable therapies in patients with T2DM in routine clinical practice, by considering its potential impact on PROs. However, high levels of missing data were observed for PRO measures throughout the study compared with those observed for clinical outcomes [22]. There are a number of potential explanations for this. For example, PROs require the patient to complete questionnaires, and this may be burdensome to the patient [48], or considered as interventional or less important than obtaining clinical information if time is constrained. Indeed, two of the PRO questionnaires used in this study were not applied in Germany due to concerns about patient burden. This ethical constraint may have resulted in patients in Germany having a perceived lighter participation burden compared with patients in other countries. Patients may also be reluctant to answer certain questions that they consider to be too personal, especially if the setting lacks privacy. Indeed, for the IWQOL-Lite subscales in CHOICE, missing data were highest for the sexual life subscale. Additionally, the high level of missing data may itself be of significance. As patients generally comply with requirements for clinical data (e.g., give blood samples as requested), there is more scope for patients to choose whether or not to answer particular questions in questionnaires, and this may lead to a self-selection bias. For example, patients with a particularly high or low HRQoL may be more compelled to complete questionnaires.

In addition to the general limitations associated with prospective observational studies, this study has some further limitations. As physicians in routine practice likely chose exenatide BID or insulin based on different patient characteristics, the data for the two cohorts cannot be directly compared or attributed to either treatment, and no statistical comparisons of the cohorts were therefore performed. The analyses in CHOICE were based on an “initiators” analysis, in which patients remained in the cohort they were placed in at baseline regardless of whether or not they changed treatment, and no adjustment of PRO data for such changes was made. PROs may also be affected over time by cognitive reframing, a natural fluctuation that can result in changes in patients’ perceptions of baseline feelings that can influence their perception of an acceptable quality of life. Additionally, social and cultural factors were not considered in this analysis.

## Conclusions

These data from the 24-month CHOICE study support those from other studies suggesting that PROs are not adversely affected, and may be improved, by the initiation of injectable therapy. As patients are taking a more active role in treatment decisions [3], and as the patient’s perception of the effects of their treatment on their quality of life may affect adherence, and therefore clinical effectiveness, PRO data can help the clinician to select the most appropriate treatment for individual patients. We believe that data such as ours enable better understanding of the psychological, as well as clinical, aspects associated with treatment selection in routine clinical practice and will become of increasing importance in shared clinical decision-making. Further research is needed to better understand psychosocial aspects that affect how patients value health and treatments [13] and to identify the most important PRO measures from an economic point of view, as well as those most associated with clinical improvements [49].

## Competing interests

Matthew Reaney, Jacek Kiljański, Carole Salaun-Martin, and Hélène Sapin are employees of Eli Lilly: Jacek Kiljański and Carole Salaun-Martin are holders of Eli Lilly shares and share options; Matthew Reaney and Hélène Sapin are not shareholders.

Chantal Mathieu is an advisory board member for Lilly Belgium. Thure Krarup is an advisory board member for Lilly Denmark.

Stephan Matthaei and Bruno Guerci have received honoraria from Eli Lilly for lectures and consultancy.

Claes-Göran Östenson has received honoraria from Eli Lilly for consultancy.

Michael Theodorakis has declared that he has no conflicts of interest.

## Authors’ contributions

All authors contributed to the study conduct/data collection, analysis, and writing of this manuscript. Matthew Reaney and Jacek Kiljański contributed to the design of the study. All authors read and approved the final manuscript.

## Authors’ information

Michael Theodorakis: Institute for Clinical Investigation, Bionian Health Sciences Cluster, 5a Stamatas Av, Drossia, GR 145 75, Athens, Greece.
